# Multicenter phase II study of trastuzumab plus S-1 alone in elderly patients with HER2-positive advanced gastric cancer (JACCRO GC-06)

**DOI:** 10.1007/s10120-017-0766-x

**Published:** 2017-09-21

**Authors:** Yutaka Kimura, Masashi Fujii, Toshiki Masuishi, Kazuhiro Nishikawa, Chikara Kunisaki, Satoshi Matsusaka, Yoshihiko Segawa, Masato Nakamura, Kinro Sasaki, Narutoshi Nagao, Yukimasa Hatachi, Yasuhiro Yuasa, Shinya Asami, Masahiro Takeuchi, Hiroshi Furukawa, Toshifusa Nakajima, on behalf of the JACCRO GC-06 Study Group, Tomono Kawase, Ryohei Kawabata, Yutaka Kimura, Tetsuya Etou, Toshiki Masuishi, Hirochika Makino, Hidetaka Ono, Yusuke Izumisawa, Chikara Kunisaki, Kazuhiro Nishikawa, Junji Kawada, Satoshi Matsusaka, Ken Shimada, Yu Sunakawa, Yoshihiko Segawa, Masato Nakamura, Hitoshi Satomura, Kinro Sasaki, Narutoshi Nagao, Katsuyuki Kunieda, Akihito Tsuji, Hironaga Satake, Yukimasa Hatachi, Hisashi Ishikura, Yasuhiro Yuasa, Hiroshi Okitsu, Shinya Asami, Takahiro Ito, Kentaro Moriichi, Masazumi Takahashi, Yasutaka Takinishi, Takashi Sekikawa, Naoki Okumura, Shigemitsu Ueyama, Noriyuki Yamamura, Dai Manaka, Sachiko Oouchi, Ryuichiro Ohashi, Tomohiko Mannami, Kazuaki Tanabe, Takeshi Shiraishi

**Affiliations:** 1 Department of Surgery, Kindai University Faculty of Medicine, Osaka-Sayama, Japan; 2 Japan Clinical Cancer Research Organization, 7F Ginza Wing Building, 1-14-5, Ginza, Chuo-ku, Tokyo, 104-0061 Japan; 3 Department of Clinical Oncology, Aichi Cancer Center Hospital, Nagoya, Japan; 4 Department of Surgery, Osaka National Hospital, Osaka, Japan; 5 Department of Surgery, Gastroenterological Center, Yokohama City University, Yokohama, Japan; 6 Department of Gastroenterology, Cancer Institute Hospital of the Japanese Foundation for Cancer Research, Tokyo, Japan; 7 Department of Medical Oncology, Saitama Medical University International Medical Center, Hidaka, Japan; 8 Aizawa Comprehensive Cancer Center, Aizawa Hospital, Matsumoto, Japan; 9 First Department of Surgery, Dokkyo Medical University Hospital, Tochigi, Japan; 10 Department of Surgery, Gifu Prefectural General Medical Center, Gifu, Japan; 11 Department of Medical Oncology, Kobe City Medical Center General Hospital, Kobe, Japan; 12 Department of Surgery, Tokushima Red Cross Hospital, Tokushima, Japan; 13 Department of Surgery, Fukuyama City Hospital, Fukuyama, Japan; 14 Department of Clinical Medicine (Biostatistics), Kitasato University School of Pharmacy, Tokyo, Japan

**Keywords:** Gastric cancer, Elderly patients, HER2-positive, S-1, Trastuzumab

## Abstract

**Background:**

S-1 plus cisplatin is a standard regimen for advanced gastric cancer (AGC) in Asia. The ToGA trial established a fluoropyrimidine plus cisplatin and trastuzumab as a standard treatment for human epidermal growth factor receptor 2 (HER2)-positive AGC. In the HERBIS-1 trial, trastuzumab combined with S-1 plus cisplatin showed promising antitumor activity in patients with HER2-positive AGC. However, cisplatin has several important drawbacks, including vomiting and renal toxicity. These disadvantages of cisplatin are prominent in elderly patients. Therefore, we conducted a prospective phase II study of trastuzumab plus S-1 without cisplatin in elderly patients with HER2-positive AGC.

**Methods:**

Patients 65 years or older who had HER2-positive AGC received S-1 orally on days 1–28 of a 42-day cycle and trastuzumab intravenously on day 1 of a 21-day cycle.

**Results:**

A total of 51 patients were enrolled. Two patients were ineligible. The full analysis set thus comprised 49 patients. The median age was 71 years (range 65–85). The confirmed response rate was 40.8% (95% CI 27.1–54.6%), and the null hypothesis was rejected. The median follow-up period was 10.6 months. Median overall survival was 15.8 months. Median progression-free survival was 5.1 months, and time to treatment failure was 4.0 months. Major grade 3 or 4 adverse events included neutropenia (12.0%), anemia (24.0%), diarrhea (10.0%), and anorexia (12.0%). There was one treatment-related death.

**Conclusions:**

Trastuzumab in combination with S-1 alone demonstrated promising antitumor activity and manageable toxic effects as well as promising survival results in elderly patients with HER2-positive AGC.

**Clinical trials registration:**

UMIN000007368.

**Electronic supplementary material:**

The online version of this article (doi:10.1007/s10120-017-0766-x) contains supplementary material, which is available to authorized users.

## Background

Gastric cancer is the second most common cause of cancer-related death worldwide [1]. The only potentially curative treatment is surgical resection. However, regional and distant recurrence often occurs after surgery. The standard treatment for advanced or recurrent gastric cancer (AGC) is chemotherapy, given in hope of prolonging survival.

A global standard regimen for AGC is a fluoropyrimidine plus a platinum compound. In Western countries, docetaxel or epirubicin is usually added to this regimen [2, 3]. In East Asia, an oral fluoropyrimidine, S-1, has become the most widely used drug for the treatment for AGC [4], and S-1 plus cisplatin is regarded as a standard regimen on the basis of the results of the phase III SPIRITS trial comparing S-1 plus cisplatin with S-1 alone for the first-line treatment of AGC [5].

Recently, the ToGA (Trastuzumab in Combination with Chemotherapy vs. Chemotherapy Alone for Treatment of HER2-Positive Advanced Gastric or Gastro-oesophageal Junction Cancer) study, an international phase III trial, evaluated trastuzumab, a humanized monoclonal antibody that blocks human epidermal growth factor receptor-2 (HER2) activation, in combination with capecitabine and cisplatin. This study demonstrated that the addition of trastuzumab provided a significant survival benefit, which was much greater in patients whose tumors highly overexpressed HER2 [immunohistochemistry (IHC), 3+ or 2+ ; fluorescent in situ hybridization (FISH) positive] [6].

A phase II trial of trastuzumab combined with S-1 plus cisplatin, a standard regimen in East Asia, was performed in patients who had AGC with high HER2 overexpression (HERBIS-1 study) [7]. The HERBIS-1 study demonstrated a high response rate (RR 68%) and prolonged survival (OS 16.0 months). Subsequently, S-1 plus cisplatin and trastuzumab became a standard regimen, as did capecitabine plus cisplatin and trastuzumab, for highly HER2-positive AGC in Japan.

However, cisplatin has several important drawbacks, including high incidences of nausea, vomiting [8], and renal toxicity [9, 10], the need for admission to receive treatment, and other adverse events negatively affecting the quality of life (QoL) of patients. Cisplatin is contraindicated in patients with poor renal function. These disadvantages of cisplatin are particularly problematic in elderly patients. Importantly, gastric cancer is a disease of aging, so the risk of it increases with advancing age.

We thus conducted this phase II study to evaluate the efficacy and safety of S-1 alone plus trastuzumab without cisplatin in elderly patients with highly HER2-positive AGC.

## Methods

### Study design

The Japan Clinical Cancer Research Organization (JACCRO) GC-06 study was a multicenter, prospective, phase II trial. The study was performed in accordance with the Declaration of Helsinki and Ethical Guidelines for Clinical Studies in Japan. The protocol was approved by the ethics committees of JACCRO and of each participating center before initiating enrollment. An Independent Data Monitoring Committee reviewed all efficacy and safety data.

### Patients

Eligible patients had to have a histologically confirmed diagnosis of gastric or esophagogastric junction adenocarcinoma, measurable disease according to the Response Evaluation Criteria in Solid Tumors (RECIST) version 1.1, and highly HER2-positive cancer as confirmed by immunohistochemistry (IHC), fluorescence in situ hybridization (FISH) (IHC 3+ or IHC2+ and FISH positive), or both. Eligible patients also had to fulfill all of the following conditions: an age of 65 years or older at the time of obtaining informed consent; an Eastern Cooperative Oncology Group (ECOG) performance status of 0, 1, or 2; adequate organ function (a leukocyte count between 3500 and 12,000/ml, a neutrophil count at least 2,000/ml, hemoglobin level at least 9.0 g/dl, a platelet count at least 100,000/ml, a serum bilirubin level of no more than 1.5 mg/dl, serum aspartate aminotransferase and alanine aminotransferase levels of no more than 100 IU; serum creatinine level less than 1.2 mg/dl, creatinine clearance no more than 50 ml/min); a left ventricular ejection fraction (LVEF) of ≥50% as measured on echocardiography or multiple gated acquisition (MUGA) scanning within 21 days before enrollment; no prior chemotherapy or radiotherapy for gastric cancer; and an expected survival of at least 3 months. Written informed consent was obtained from all patients.

### Treatment

Patients received S-1 (80–120 mg per day) orally on days 1–28 of a 42-day cycle and trastuzumab (first dose 8 mg/kg; second dose onward 6 mg/kg) intravenously on day 1 of a 21-day cycle (the criteria for withholding, resuming, and dose reduction of S-1 and trastuzumab, and the modification of treatment with S-1 are shown in the table in the “Electronic supplementary material”).

### Outcomes

The primary endpoint of this study was the response rate (RR). The secondary endpoints were overall survival (OS), progression-free survival (PFS), time to treatment failure (TTF), and safety. Tumors were measured every 6 weeks by the investigators at each participating center until the onset of progressive disease. An extramural review committee assessed all images obtained in the study according to the RECIST. The OS was defined as the time from the date of enrollment to the date of death from any cause. PFS was defined as the time from the date of enrollment to the date of disease progression or death from any cause. TTF was defined as the time from the date of enrollment to the date when the treating physician decided to discontinue treatment for any reason. Physical examinations and blood tests were mandatory before each course of treatment, and LVEF was assessed every 3 months during treatment. Adverse events were evaluated according to the National Cancer Institute Common Terminology Criteria for Adverse Events, version 4.0.

### Statistical analysis

The reported RR of S-1 alone in elderly patients with AGC was 14.3–21.7% [11–13] and the RR of fluoropyrimidine plus cisplatin was reported 35% by ToGA trial. Then, the required sample size was estimated on the basis of a threshold RR of 20% and an expected RR of 35%, 80–90% power, and an alpha value of 0.1 (one-sided) using the binomial test. Due to eventual ineligible patients, the target sample size was determined to be at least 40 patients at a power of 80%. Efficacy was evaluated in all patients who received the study treatment. We used the Kaplan–Meier method to estimate survival curves and Greenwood’s formula to calculate 95% confidence intervals (CI) for survival rates. Statistical analyses were conducted with SAS software, version 9.2.

## Results

### Patient disposition and characteristics

Patients were enrolled from March 2012 through February 2014. The last follow-up analysis was conducted in March 2016. Forty patients were enrolled before completion of the planned accrual period of 2 years, increasing the power of this study from 80 to 90%. Finally, 51 patients were enrolled in the study within 2 years at 24 centers in Japan. However, two patients were ineligible because they lacked measurable lesions or could not receive the protocol treatment. A total of 50 patients, including the patient with no measurable lesion, were included in safety analyses, and the full analysis set comprised 49 patients (actual power 87%). The characteristics of the 49 patients are shown in Table 1. The median age was 71 years (range 65–85), and the ECOG performance status was 0 in 32 patients, 1 in 14 patients, and 2 in 3 patients. Thirty-seven patients (76%) had differentiated adenocarcinoma. Forty-one patients (84%) had unresectable lesions, and 8 patients (16%) had recurrent disease. The most frequent site of metastasis was lymph nodes (78%), followed by the liver (45%), lung (14%), and peritoneum (14%). The proportions of IHC 3+ and IHC 2+/FISH-positive tumors were 71 and 29%, respectively.

**Table 1:** Patient characteristics

Characteristics	*n* = 49
Age (years)	
Median	71
Range	65–85
Gender	
Male	36 (73.5%)
Female	13 (26.5%)
ECOG PS	
0	32 (65.3%)
1	14 (28.6%)
2	3 (6.1%)
Histological type	
Differentiated	37 (75.5%)
Undifferentiated	12 (24.5%)
Previous gastrectomy	
No	36 (73.5%)
Yes	13 (26.5%)
Unresectable/recurrent	
Unresectable	41 (83.7%)
Recurrent without adjuvant chemotherapy	5 (10.2%)
Recurrent with adjuvant chemotherapy	3 (6.1%)
Metastatic sites	
Lymph nodes	38 (77.6%)
Liver	22 (44.9%)
Lung	7 (14.3%)
Peritoneum	7 (14.3%)
HER2 status	
IHC3+	35 (71.4%)
IHC2+ and FISH-positive	14 (28.6%)

*ECOG PS* Eastern Cooperative Oncology Group performance status, *IHC* immunohistochemistry

### Efficacy

All patients were treated on an outpatient basis. The median number of treatment cycles of S-1 was 3.0 (range 1–29), and the median relative dose intensity was 90.4% for S-1 and 100% for trastuzumab. At the time of analysis, one patient was still receiving treatment, and all other patients had discontinued treatment. The main reason for discontinuation of treatment was progressive disease (37 patients). Only 5 patients with thrombocytopenia, pneumothorax, ileus, hand-foot syndrome and febrile neutropenia discontinued treatment, and 2 patients with para-aortic lymph node metastasis after 2–3 cycles of treatment underwent surgery because of a significant response.

**Fig. 1:**
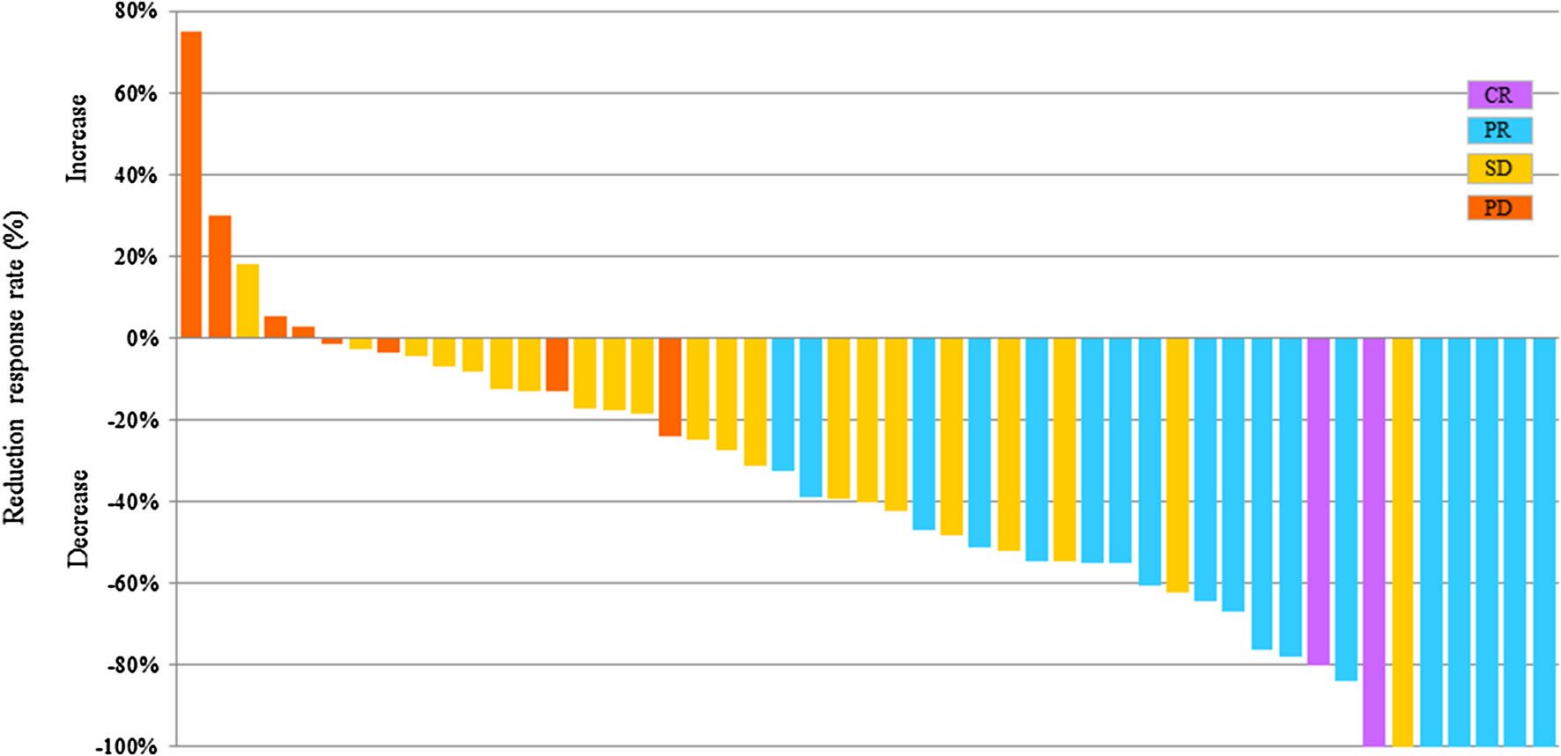
A waterfall plot showing the confirmed best overall response for each patient

**Fig. 2:**
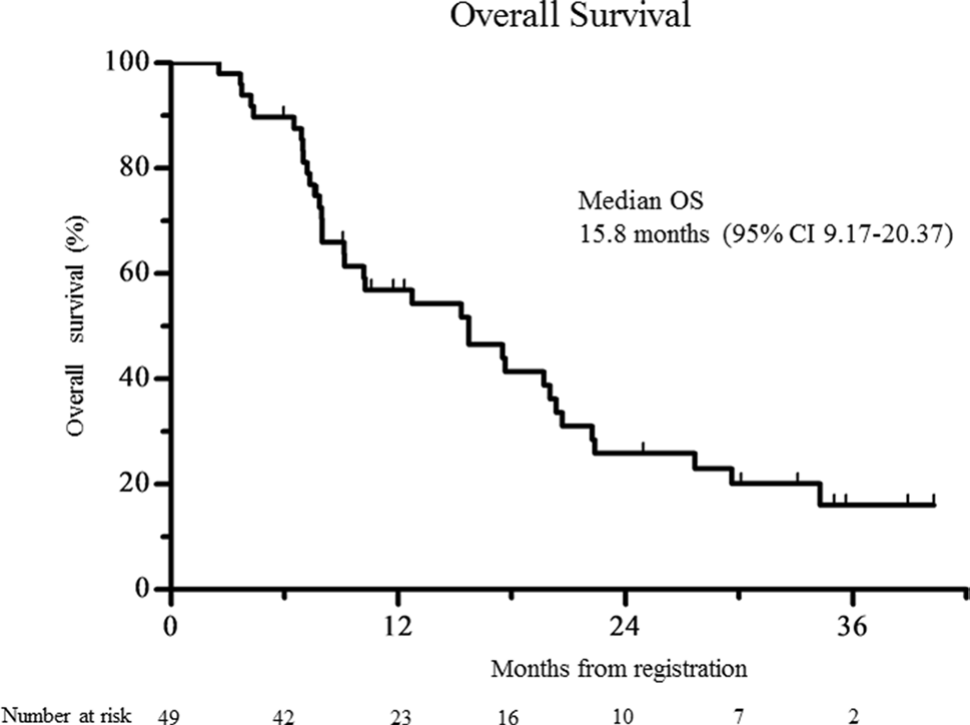
The Kaplan–Meier overall survival

**Fig. 3:**
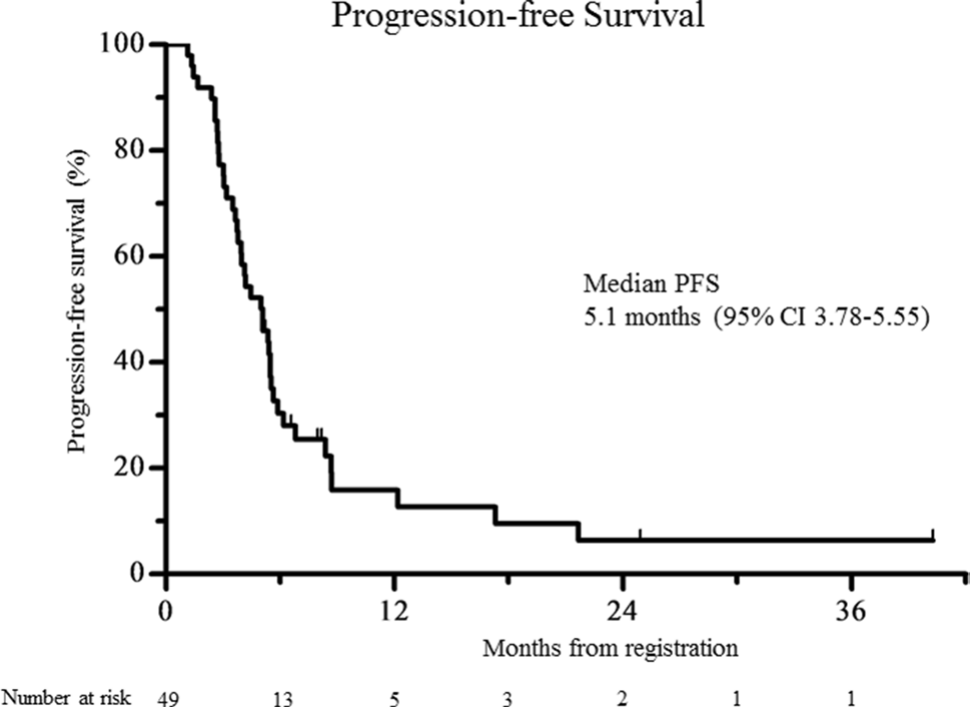
The Kaplan–Meier progression-free survival

The confirmed RR [complete response (CR), 2 patients; partial response (PR), 18 patients; stable disease (SD), 21 patients; progressive disease (PD), 8 patients] was 40.8% (95% CI 27.1–54.6%; 80% CI 31.8–49.8%); therefore, the null hypothesis for the primary end point (RR = 20%) was rejected. The confirmed RR was 40.5% (95% CI 24.7–56.4%) in the patients with differentiated cancer (*n* = 37) and 41.7% (95% CI 13.8–69.6%) in the patients with undifferentiated cancer (*n* = 12). Among the 20 patients with CR or PR, the median time to response and duration of response were 43 days (range 27–120 days) and 134 days (range 43–1152 days), respectively. The disease control rate (i.e., the proportion of patients who had CR, PR, or SD) was 83.7% (95% CI 73.3–94.0%). A waterfall plot of the confirmed best overall response for each patient is shown in Fig. 1. The median duration of follow-up for the 49 patients at the time of analysis (March 2016) was 10.6 months. The median OS was 15.8 months (95% CI 9.2–20.4), and the 1-year OS rate was 56.9% (95% CI 41.4–69.7%; Fig. 2). The median PFS was 5.1 months (95% CI 3.8–5.6), and the 1-year PFS rate was 15.9% (95% CI 6.5–29.1%; Fig. 3). The median TTF was 4.0 months (95% CI 3.1–5.4).

There was no statistically significant difference between patients with IHC3+ (*n* = 35) and IHC2+/FISH-positive (*n* = 14) status, but the RR of IHC3+ was higher than that of IHC2+ and FISH-positive status (48.6 vs. 21.4%, *p* = 0.11), and overall survival was longer in IHC3+ than in IHC2 and FISH positive status (17.5 vs. 8.0 months, *p* = 0.36).

### Safety

All adverse events are summarized in Table 2. Among the hematological adverse events, the rates of grade 3 or higher neutropenia and anemia were 12 and 24%, respectively. The most frequent common nonhematological toxicity was anorexia (any grade 66%; grade 3 or higher 12%). There was no grade 3 or higher nausea or vomiting. The incidence of elevations of the creatinine level of any grade was only 10%, and there was no grade 3 or higher renal toxicity. Grade 3 infusion-related reactions occurred in 1 patient (2%). Heart failure did not occur in any patient. One patient was evaluated to have died from treatment-related causes. This patient was an 80-year-old man with peritoneal metastasis from AGC. There was no severe adverse event during the prior four courses of S-1 plus trastuzumab. Nine days after the fifth dose of trastuzumab, he was found dead at his home. The reason for sudden death was unknown, but we considered the death to be treatment-related because the patient died within 30 days after the last infusion of trastuzumab.

**Table 2:** Adverse events

Event	*n* = 50				Any (%)	Grade 3–4 (%)
	1	2	3	4		
Leukopenia	10	10	5	0	50.0	10.0
Neutropenia	6	5	6	0	34.0	12.0
Febrile neutropenia	–	–	1	0	2.0	2.0
Anemia	6	12	12	0	60.0	24.0
Thrombocytopenia	18	1	1	0	40.0	2.0
AST increased	16	3	0	0	38.0	0.0
ALT increased	10	0	0	0	20.0	0.0
Creatinine increased	5	0	0	0	10.0	0.0
Bilirubin increased	7	3	2	0	24.0	4.0
Hypoalbuminemia	5	14	3	0	44.0	6.0
Diarrhea	16	4	5	0	50.0	10.0
Oral mucositis	10	4	4	0	36.0	8.0
Anorexia	5	22	6	0	66.0	12.0
Fatigue	10	4	1	–	30.0	2.0
Nausea	14	8	–	–	44.0	0.0
Vomiting	11	4	0	0	30.0	0.0
Infusion-related reaction	0	0	1	0	2.0	2.0
Hypertension	8	9	2	0	38.0	4.0
Palmar-plantar erythrodysesthesia syndrome	4	2	2	–	16.0	4.0

## Discussion

Cancer in the elderly is rapidly increasing worldwide. More than 60% of patients with gastric cancer are older than 65 years at the time of diagnosis, and about one-third are older than 75 years [14]. Systemic chemotherapy has improved OS and the QoL as compared with supportive care alone in patients with AGC [15–17]. The chronologic age alone is an inadequate reason for avoiding the use of effective cancer treatment that can improve QoL or OS. However, cytotoxic agents such as cisplatin often negatively affect the QoL of elderly patients. A subgroup analysis of the SPIRITS trial reported that the HR of elderly patients was worse than that of younger patients (younger than 60 years of age: HR 0.75; 60–69 years of age: HR 0.98; 70–74 years of age: HR 0.95) [5]. On the other hand, a subgroup analysis of the ToGA study reported that the HR was 0.84 in patients younger than 60 years, as compared with 0.66 in patients 60 years or older, and adding trastuzumab to chemotherapy did not increase adverse events [6]. Therefore, we hypothesized that cisplatin would not prolong survival in elderly patients with tumors overexpressing HER2, and that combining trastuzumab with chemotherapy should be considered. This multicenter phase II study demonstrated that a fluoropyrimidine plus trastuzumab without cisplatin has favorable efficacy and lower toxicity in elderly patients with HER2-positive AGC.

In our study, the RR and disease control rate (DCR) were 40.8 and 83.7%, respectively. The RR in our study was lower than those in the ToGA (47%) and HERBIS-1 (68%) studies. However, the DCR was comparable to those in the ToGA (79%) and HERBIS-1 (94%) studies. The OS of patients who received a fluoropyrimidine plus trastuzumab without cisplatin was 15.8 months. This survival benefit was similar to those in the HERBIS-1 (16.0 months) and ToGA (highly overexpressing HER2-positive patients, 16.0 months) studies. On the other hand, the PFS of patients who received a fluoropyrimidine plus trastuzumab without cisplatin was 5.1 months, which was much shorter than those in the HERBIS-1 (7.8 months) and ToGA (6.7 months) studies. We attribute the long survival time after the onset of disease progression to the fact that 65.3% of patients received second-line chemotherapy, among whom 59.4% consecutively received trastuzumab. The toxicities of a fluoropyrimidine plus trastuzumab without cisplatin were very mild, as we had expected. In our study, the incidence of grade 3 or higher neutropenia was 12% (HERBIS-1 36%; ToGA 27%), febrile neutropenia was 2% (HERBIS-1 4%; ToGA 5%), grade 3 or higher vomiting was 0% (HERBIS-1 6%; ToGA 6%), and a grade 3 or higher increased creatinine level was 0% (HERBIS-1 6%; ToGA 1%). These mild toxicities of a fluoropyrimidine plus trastuzumab without cisplatin as the first-line treatment for HER2-positive AGC may have had a positive effect on the introduction of second-line treatment. At the start of the second-line treatment, ECOG performance status was 0 in 17 patients, 1 in 15 patients, 2 in 2 patients, and 3 in 1 patient. The incidence of grade 3 or higher anemia in our study was 24% (HERBIS-1 15%; ToGA 12%), which was significantly higher than that in the ToGA study (*p* = 0.003). Anemia is easy controlled, but should be effectively managed in elderly patients who receive S-1. All patients could receive treatment on an outpatient basis without being admitted to the hospital. We believe that this factor also contributed substantially to the QoL of the elderly patients.

This study had some limitations. Although the PFS was shorter and the OS was almost the same in HERBIS-1 compared to the ToGA study, no details regarding post-progression treatment were provided for HERBIS-1.

## Conclusion

We conclude that trastuzumab plus S-1 alone without cisplatin is an effective and safe treatment that can prolong survival in elderly patients with HER2-positive AGC.

## Electronic supplementary material

Below is the link to the electronic supplementary material.

Supplementary material 1 (DOCX 223 kb)
