# Supplementary material for: Multicenter phase II study of trastuzumab plus S-1 alone in elderly patients with HER2-positive advanced gastric cancer (JACCRO GC-06)
Source: Gastric Cancer. 2017 Sep 21;21(3):421–7. doi: 10.1007/s10120-017-0766-x (PMC5906490; doi:10.1007/s10120-017-0766-x)
Supplement: Supplementary file 1 — Supplementary material 1 (DOCX 223 kb) [file 10120_2017_766_MOESM1_ESM.docx]

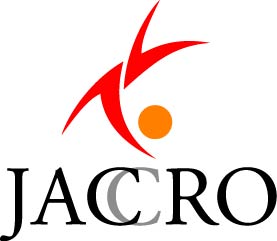


A Phase II Study of Combination Therapy With TS-1 and Trastuzumab in Patients 65 Years or Older Who Have HER2-positive, Advanced or Recurrent Gastric Cancer

JACCRO GC-06

Protocol

Study representative: Hiroya Takiuchi

Osaka Medical College Hospital

Substitute study representative: Masashi Fujii

Surugadai Nihon University Hospital

Ver 1.0: December 13, 2011

Ver 1.1: July 24, 2012

Japan Clinical Cancer Research Organization

# 0. Study outline

## 0.1. Study title

A phase II study of combination therapy with TS-1 and trastuzumab in patients 65 years or older who have HER2-positive, advanced or recurrent gastric cancer

## 0.2. Study objective

To study the efficacy and safety of combination therapy with TS-1 and trastuzumab in patients 65 years or older who have HER2-positive, measurable, advanced or recurrent gastric cancer.

## 0.3. Study type

A multicenter, collaborative, phase II study using a central registration system

## 0.4. Subjects

Patients aged 65 years or older who have advanced or recurrent gastric cancer with a diagnosis of HER2-positive (IHC3C+, or IHC2+ and FISH-positive) primary or metastatic lesions.

## 0.5. Eligibility criteria

Patients who meet all of the following criteria are eligible.

1) Patients with unresectable or recurrent gastric cancer histologically confirmed to be adenocarcinoma. Esophagogastric cancer that is centrally located within 2 cm from the superior and inferior margins of the esophagogastric junction is acceptable.

2) Patients with measurable lesions according to RECIST version 1.1

3) Patients with a diagnosis of HER2-positive (IHC3+, or IHC2+ and FISH-positive) cancer in the primary lesion or metastatic lesions

4) Patients 65 years or older at the time of obtaining informed consent

5) Patients with a performance status (ECOG) of 0 to 2

6) Patients who have not received chemotherapy or radiotherapy for advanced or recurrent gastric cancer. However, patients in whom more than 6 months have elapsed from the time of completing adjuvant chemotherapy to the time of recurrence are eligible.

7) Patients with adequate main organ functions on examinations performed within 14 days before enrollment (examinations performed on the same day of the week 2 weeks before enrollment are acceptable) as defined by the following variables:

① White blood cell count: 3.5 x 10^3^/µL or higher and less than 12 x 10^3^/µL

② Neutrophil count: 2000/µL or higher

③ Platelet count: 100 × 10^3^/µL or higher

④ Serum hemoglobin level: 8.0 g/dL or higher

⑤ AST level: less than 100 IU/L

⑥ ALT level: less than 100 IU/L

⑦ Serum bilirubin level: less than 1.5 mg/dL

⑧ Serum creatinine level: 1.2 mg/dL or less

⑨ Creatinine clearance: 50 mL/min or higher^※1※2^

※1 The Cockcroft-Gault formula is used. However, if actual measurement values are available, the actual values are given priority.

[Cockcroft-Gault formula: CCr = body weight (kg) × (140-age)/(72 × serum creatinine level (mg/dL). For females, the obtained value is multiplied by a constant of 0.85.)

※2 If the creatinine clearance level is 50 mL/min or higher and less than 60 mL/min, the initial daily dose of TS-1 is decreased by one level in accordance with "Table 7.2.1. Initial daily dose of TS-1."

8) Patients with a left ventricular ejection fraction (LVEF) of 50% or higher as calculated on echocardiography or a MUGA scan within 21 days before enrollment

9) Patients with no clinically problematic abnormalities on 12-lead electrocardiography performed within 21 days before enrollment

10) Patients in whom oral intake is possible

11) Patients who are expected to survive for at least 3 months from the date of enrollment

12) Patients who were notified of the name of their disease and gave informed consent in writing

## 0.6. Exclusion criteria

Patients who meet any of the following criteria will be excluded from the study.

1) Patients with synchronous double cancers or metachronous double cancers with a disease-free interval of 5 years or less (However, lesions of intramucosal cancer are not regarded to be active double cancers.)

2) Patients with massive accumulation of cancerous body fluid (pleural effusion, ascites, pericardial effusion)

3) Patients with distinct brain metastasis

4) Patients with bleeding from gastric cancer or peptic ulcers

5) Patients with infection who have a fever of 38.0°C or higher

6) HBs antigen-positive patients

7) Patients with a previous history of congestive heart failure

8) Patients with angina pectoris requiring drug therapy

9) Patients with electrocardiographically confirmed intramural myocardial infarction

10) Patients with poorly controlled hypertension (systolic blood pressure >180 mmHg or diastolic blood pressure >100 mmHg)

11) Patients with clinically distinct cardiac valvular disease

12) Patients with high-risk, uncontrollable arrhythmias

13) Patients with serious complications (interstitial pneumonia or pulmonary fibrosis, heart failure, renal failure, liver failure, poorly controlled diabetes mellitus, etc.)

14) Patients with dyspnea at rest

15) Patients with diarrhea (4 times or more per day or watery stool)

16) Patients in whom TS-1 and trastuzumab are contraindicated (refer to the latest package insert)

17) Patients with a previous history of hypersensitivity to TS-1 or trastuzumab

18) Patients who are receiving flucytosine

19) Patients who are continuously receiving phenytoin or warfarin potassium

20) Patients who are receiving long-term or high-dose steroid therapy

21) Pregnant women or women who may be pregnant and men who wish their partner to become pregnant

22) Patients who are judged by the study director or attending physician to be ineligible for the study.

## 0.7. Study design

### 0.7.1. Schema

Patients 65 years or older who have HER2-positive (IHC3+, or IHC2+ and FISH-positive), advanced or recurrent gastric cancer and a performance status of 0 to 2 and have not previously received chemotherapy

↓

Informed consent obtained

↓

Patient enrollment

↓ : within 14 days

Protocol treatment is begun

Figure 0.7.1. Study schema

0.7.2. Definition and enrollment of HER2-positive patients in this study

In this study, the study group comprises patients with HER2-positive gastric cancer (65 years or older). Therefore, whether tumors are HER2 positive must be evaluated in each hospital before enrollment in the study. The details are described below. Patients evaluated to be IHC3+, or IHC2+ and FISH-positive in each hospital who meet the inclusion criteria of the study will be enrolled.

0.7.3. Precautions when HER2 testing is performed in your hospital

In each hospital, HER2 testing should be performed in accordance with appropriate procedures, referring to the latest guidelines for HER2 testing of gastric cancer and the package insert of extracorporeal diagnostic pharmaceuticals.

Examples of the methods and procedures for measuring HER2 positivity in gastric cancer are shown in the Appendix.

## 0.8. Dosage and treatment schedule for combination therapy with TS-1 plus trastuzumab

The protocol treatment is started within 14 days after patient enrollment by the FLADS® system. (The same day of the week 2 weeks after the date of enrollment is acceptable.)

Trastuzumab

Trastuzumab

Trastuzumab

Trastuzumab

Day 70

Day 84

Day 64

（Course 2, Day 22）

Day 1

Day 22

Day 28

Day 43

（Course 2, Day 1）

Rest

TS-1

Rest

TS-1

Course 1

Course 2

Figure 0.8. Treatment schedule for combination therapy with TS-1 plus trastuzumab

## 0.8.1. Treatment regimens of TS-1

### 1) Treatment with TS-1 is started after breakfast on day 1 according to “Table 0.8.1. Daily dose of TS-1.” TS-1 is given orally twice daily after meals (breakfast and dinner) until after dinner on day 28, followed by a 14-day rest.

2) Even if treatment is withheld during a course or patients forget to take their medication, treatment is not performed after day 29. However, if treatment cannot be given after breakfast on day 1, treatment is started after dinner and is continued until after breakfast on day 29.

Table 0.8.1. Daily dose of TS-1

| Body-surface area^※1^ | Daily dose (tegafur equivalent) | |
| --- | --- | --- |
|  | Creatinine clearance  (≥60 mL/min) | Creatinine clearance  (≥50 mL/min and <60 mL/min) |
| <1.25 m^2^  ≥1.25 m^2^ to <1.5 m^2^  ≥1.5 m^2^ | 80 mg/day  100 mg/day  120 mg/day | 60 mg/day^※2^  80 mg/day  100 mg/day |

※1: Fujimoto formula: body-surface area (m^2^) = body weight (kg)^0.444^ × height (cm)^0.663^ × 0.008883

※2: morning, 40 mg; evening, 20 mg

### 0.8.2. Treatment regimens of trastuzumab

The initial dose of trastuzumab is 8 mg/kg (body weight), and the second and subsequent doses are 6 mg/kg (body weight). Trastuzumab is given as a continuous intravenous infusion on days 1 and 22. The initial dose is administered as an intravenous infusion over the course of 90 minutes or longer. If tolerance is good, the infusion time of the second and subsequent doses can be shortened to 30 minutes.

### 0.8.3. Treatment period

Treatment is continued until patients meet "8. Criteria for discontinuing the protocol treatment."

## 0.9. Evaluated variables

Primary endpoint: Response rate

Secondary endpoints: Overall survival

Progression-free survival

Time to treatment failure

Incidence and severity of adverse events

## 0.10. Target number of patients

Target number of patients: 40 patients

## 0.11. Study period

Patient enrollment period: March 2012 through February 2014

Follow-up period: 2 years after enrollment of the final patient

Study period: March 2012 through February 2016

# 7. Protocol treatment

## 7.1. Criteria for starting the protocol treatment

1) The protocol treatment is started within 14 days after patients are enrolled by the FLADS® system. (Same day of the week 2 weeks after the day of enrollment is acceptable.)

2) It is confirmed that laboratory values and clinical symptoms on the day before starting treatment or the day of treatment meet all conditions of “Table 7.1. Criteria for starting the protocol treatment.”

3) If patients do not meet the conditions of "Table 7.1. Criteria for starting the protocol treatment" within 14 days after enrollment and the protocol treatment cannot be started, they are regarded as patients discontinuing the study before treatment.

Table 7.1. Criteria for starting the protocol treatment

| Variable | Criteria |
| --- | --- |
| Neutrophil count | ≥2000/µL |
| Platelet count | ≥100 × 10^3^/µL |
| Hemoglobin | ≥8.0 g/dL |
| AST and ALT | <100 IU/L |
| Serum bilirubin | <1.5 mg/dL |
| Serum creatinine | ≤1.2 mg/dL |
| Creatinine clearance | ≥50 mL/min  If the creatinine clearance is ≥50 mL/min and <60 mL/min, the initial dose of TS-1 is decreased by one level, according to "Table 7.2.1. Daily dose of TS-1." |
| Body temperature | <38.0°C |
| Gastrointestinal symptoms (diarrhea, stomatitis, nausea, vomiting, anorexia) | ≤Grade 1 |
| Palpitations, shortness of breath, tachycardia | No symptoms |

## 7.2. Protocol treatment schedule

1) Treatment with TS-1 is started after confirming that the patient meets "Table 7.5.1 Criteria for starting treatment with TS-1 (second course onward)" during each course of treatment. The day of starting treatment with TS-1 is designated as day 1 of each course.

2) In accordance with "Table 7.2.1. Daily dose of TS-1," the dose of TS-1 determined according to the body-surface area (Fujimoto formula) and creatinine clearance is given orally twice daily (after breakfast and dinner) for 28 consecutive days from day 1 to 28, followed by a 14-day rest.

3) Treatment with trastuzumab is performed according to "Table 7.5.2 Criteria for continuing treatment with trastuzumab."

4) Trastuzumab is given in an initial dose of 8 mg/kg (body weight). The second and subsequent doses are 6 mg/kg (body weight). Trastuzumab is given as a continuous intravenous infusion on days 1 and day 22 (at a 3-week interval).

5) Treatment is repeated until the patient meets any of the criteria for discontinuing the protocol treatment.

6) The protocol treatment can be given earlier or delayed because of holidays or the patient’s circumstances other than adverse events.

### 7.2.1. Treatment regimens of TS-1

1) In accordance with "Table 7.2.1. Daily dose of TS-1," TS-1 is started after breakfast on day 1 and is given orally twice daily (after breakfast and dinner) until after dinner on day 28, followed by a 14-day rest.

2) Even if treatment is withheld or patients forget to take their medication, treatment is not given after day 29. However, if treatment cannot be given after breakfast on day 1, treatment is started after dinner and continued until after breakfast on day 29.

### 7.2.2. Treatment regimens of trastuzumab

1) Because infusion reactions are highly likely to occur at the time of initial treatment with trastuzumab, patients should receive treatment during "hospitalization" whenever possible. If the patient is treated on an outpatient basis, the patient should be able to immediately receive treatment on the occurrence of any abnormality.

2) The initial dose of trastuzumab is 8 mg/kg (body weight), and the second and subsequent doses are 6 mg/kg (body weight), administered as a continuous intravenous infusion on day 1 and day 22 (3-week interval) of each course of treatment. The initial dose is given over the course of at least 90 minutes. If tolerance to the initial dose is favorable, the infusion time of the second and subsequent doses can be shortened to up to 30 minutes.

3) If the body weight measured before treatment increases or decreases by 10% or more after initial treatment, the dose should be recalculated.

4) In accordance with the package insert of trastuzumab, trastuzumab is dissolved in the attached Japanese Pharmacopeia water for injection to a trastuzumab concentration of 21 mg/mL. Then, the required amount is removed by a glass syringe and is immediately dissolved in 250 mL of Japanese Pharmacopeia physiological saline solution, which is given as a continuous intravenous infusion.

## 7.3. Treatment period

Treatment is continued until the patient meets "8. Criteria for discontinuing the protocol treatment.

## 7.4. Criteria for withholding and resuming the protocol treatment

### 7.4.1. Criteria for withholding and resuming treatment with TS-1

### 1) If the patient meets any of the conditions of "Table 7.4.1.1. Criteria for withholding treatment with TS-1" during treatment with TS-1, TS-1 is withheld.

2) After confirming that a patient in whom TS-1 was withheld meet the conditions of "Table 7.4.1.2. Criteria for resuming treatment with TS-1 within a course," treatment with TS-1 is resumed.

3) Within the same course of treatment, TS-1 is not given after day 29.

4) If treatment with TS-1 is withheld because of adverse events other than "Table 7.4.1.1. Criteria for withholding treatment with TS-1," and the study director, etc. considers it possible administer treatment after alleviation or recovery, treatment can be resumed.

Table 7.4.1.1. Criteria for withholding treatment with TS-1

| Variable | Criteria |
| --- | --- |
| Neutrophil count | <1000/µL |
| Platelet count | <75 × 10^3^/µL |
| Serum creatinine | >1.2 mg/dL |
| Infection | Fever of ≥38.0°C with suspected infection |
| Febrile neutropenia | ≥Grade 3 |
| Gastrointestinal symptoms (diarrhea, stomatitis, nausea, vomiting, anorexia) | ≥Grade 2 |
| Other nonhematologic conditions | ≥Grade 3 |

If the study director judges that treatment is difficult to continue because of adverse events that do not meet the criteria described above, treatment can be withheld.

Table 7.4.1.2. Criteria for resuming treatment with TS-1 within a course

| Variable | Criteria |
| --- | --- |
| Neutrophil count | ≥1500/µL |
| Platelet count | ≥75 × 10^3^/µL |
| Serum creatinine | ≤1.2 mg/dL |
| Infection | No fever of ≥38.0°C with suspected infection |
| Gastrointestinal symptoms (diarrhea, stomatitis, nausea, vomiting, anorexia) | ≤Grade 1 |

### 7.4.2. Criteria for withholding and resuming treatment with trastuzumab

1) If a patient has any of the conditions described in "Table 7.4.2.1. Measures taken on the onset of adverse events caused by trastuzumab” while receiving trastuzumab, treatment with trastuzumab is withheld or discontinued, and subsequent action is taken in accordance with the corresponding description.

2) If the patient has palpitations, shortness of breath, tachycardia, or a fever of 38.0°C or higher, recover is awaited and treatment is performed.

3) Treatment is administered after confirming that at least 7 days have elapsed since treatment with trastuzumab in the previous course.

Table 7.4.2.1. Measures taken on the onset of adverse events caused by trastuzumab

|  | Description of event | Measures during treatment with trastuzumab |
| --- | --- | --- |
| Infusion reactions | Mild or moderate | If the reactions described to the left occur during continuous intravenous infusion of trastuzumab, the continuous intravenous infusion is discontinued. Antipyretic analgesics, antihistamines, etc. are administered. After symptoms resolve, treatment can be resumed at a slower infusion rate. |
|  | Severe  (anaphylactic symptoms, tachypnea, bronchospasm, hypotension, hypoxemia, etc.) | Continuous intravenous infusion of trastuzumab is immediately discontinued. Subsequently, retreatment is not performed. |
| Congestive heart failure | Patients with clinical signs and symptoms suggestive of congestive heart failure in whom decreased LVEF is definitively diagnosed on chest radiography and MUGA scanning or electrocardiography | Treatment with trastuzumab is discontinued and is not resumed subsequently. |
| Cardiac disease | Patients with cardiac disease corresponding to NYHA III/IV | Treatment with trastuzumab is discontinued. Subsequently, retreatment is not performed. |
|  | LVEF | Treatment is performed according to the algorithm for treatment with trastuzumab in patients with LVEF. |

Table 7.4.2.2. New York Heart Association (NYHA) classification

| NYHA classification | |
| --- | --- |
| Stage I | Cardiac disease, but no symptoms and no limitation of ordinary physical activity. |
| Stage II | Patients with cardiac disease who have mild to moderate limitation of ordinary physical activity. No symptoms at rest, but ordinary physical activity results in fatigue, palpitations, dyspnea, or angina. |
| Stage III | Patients with cardiac disease who have marked limitation of ordinary physical activity. No symptoms at rest. However, walking on a flat surface or less than ordinary physical activity causes fatigue, leading to symptoms. |
| Stage IV | Patients with cardiac disease in whom symptoms occur even during very mild physical activity. Symptoms of heart failure or angina pectoris may occur even at rest. |

LVEF ≥45％

LVEF <45％

Trastuzumab

continued

LVEF ≥40% to <45%

LVEF ≤39％

Treatment postponed

LVEF reevaluated within 3 weeks after final dose

Decrease of ≥10 points from baseline

Decrease of <10 points from baseline

Treatment postponed

LVEF reevaluated within 3 weeks

Trastuzumab

continued

LVEF ≥40% to <45% with decrease of <10 points from baseline, or LVEF ≥45%

LVEF ≤39％ or LVEF ≥40% to <45% with decrease of ≥10 points from baseline

LVEF ≥40% to <45% with decrease of <10 points from baseline, or LVEF ≥45%

LVEF ≤39％ or LVEF ≥40% to <45% with decrease of ≥10 points from baseline

Trastuzumab

withdrawn

Trastuzumab

withdrawn

Trastuzumab

resumed

Trastuzumab

resumed

Figure 7.4.2. Algorithm for treatment with trastuzumab according to LVEF

## 7.5. Criteria for starting each course of treatment (course 2 onwards)

1) After confirming that laboratory values and clinical symptoms 1 day before the anticipated day of starting each course of treatment (course 2 onwards) or on the day of treatment meet all conditions of "Table 7.5.1. Criteria for starting a course of treatment with TS-1 (course 2 onwards)" and "Table 7.5.2. Criteria for continuing treatment with trastuzumab," the course of treatment with TS-1 and trastuzumab is begun.

2) If a patient meets the conditions of "Table 7.5.2. Criteria for continuing treatment with trastuzumab," but does not meet the conditions of "Table 7.5.1. Criteria for starting a course of treatment with TS-1 (course 2 onwards)," only trastuzumab is administered.

3) If a patient meets the conditions of "Table 7.5.1. Criteria for starting a course of treatment with TS-1 (course 2 onwards)," but does not meet the conditions of "Table 7.5.2. Criteria for continuing treatment with trastuzumab," a course of treatment with only TS-1 is begun.

4) If the next course of TS-1 cannot be administered for more than 28 days after the final day of treatment with TS-1, the protocol treatment is discontinued.

Table 7.5.1. Criteria for starting a course of treatment with TS-1 (course 2 onwards)

| Variable | Criteria for starting treatment course (course 2 onwards) |
| --- | --- |
| Neutrophil count | ≥1500/µL |
| Platelet count | ≥75 × 10^3^/µL |
| Serum creatinine | ≤1.2 mg/dL |
| Infection | No fever (≥38.0°C) with suspected infection |
| Gastrointestinal symptoms (diarrhea, stomatitis, nausea, vomiting, anorexia) | ≤Grade 1 |
| Previous course of treatment with trastuzumab^※^ | Treatment is given after at least 7 days have elapsed since treatment with trastuzumab in the previous course.  (e.g., If trastuzumab was given on day 22, the next course is begun on day 29 or subsequently.) |

※ To administer trastuzumab at the start of the treatment course (day 1), continuous treatment with trastuzumab (treatment within 1 week after the previous dose) should be avoided.

Table 7.5.2. Criteria for continuing treatment with trastuzumab

| Variable | Criteria for continuing treatment (course 2 onwards) |
| --- | --- |
| Palpitations, shortness of breath, tachycardia | No symptoms |
| Hypertension | Absence of poorly controlled hypertension (systolic blood pressure >180 mm Hg or diastolic blood pressure >100 mm Hg) |
| LVEF | In accordance with the algorithm for treatment with trastuzumab according to LVEF |
| Body temperature | <38.0°C |

## 7.6. Criteria for dose reduction at the time of resuming treatment with TS-1

1) If a patient meets the conditions of "Table 7.4.1.1. Criteria for withholding treatment with TS-1" within a course of treatment, treatment with TS-1 is withheld.

2) If a patient meets the conditions of "Table 7.6.1. Criteria for dose reduction at the time of resuming treatment with TS-1" within a course of treatment, the dose of TS-1 at the time of resuming treatment is decreased in accordance with "Table 7.6.1 Criteria for dose reduction at the time of resuming treatment with TS-1" and "Table 7.6.2. Reduced dose levels of TS-1."

3) When the study director or attending physician judges that treatment should be withheld, treatment with TS-1 can be withheld, or the dose of TS-1 can be decreased, even if the patient does not meet the conditions of "Table 7.4.1.1. Criteria for withholding treatment with TS-1."

4) The dose of TS-1 can be decreased even during the same course of treatment.

5) If adverse events requiring dose reduction occur even after the dose of TS-1 has been decreased to the lowest level, the protocol treatment is discontinued.

6) If the study director or attending physician judges that there is no problem with safety in patients in whom dose reduction was performed because factors such as adverse events, the dose can be increased to the initial dose.

7) Even if treatment with trastuzumab is discontinued, TS-1 should be continuously administered unless the patient meets "8. Criteria for discontinuing protocol treatment."

Table 7.6.1. Criteria for dose reduction at the time of resuming treatment with TS-1

| Variable | Criteria for dose reduction | |
| --- | --- | --- |
|  | Status at treatment withdrawal | Presence or absence of dose reduction at time of resuming treatment |
| Neutrophil count | ≥500/µL  <1000/µL | Dose not decreased |
|  | <500/µL | Dose decreased by one level |
| Febrile neutropenia | ≥Grade 3 | Dose decreased by one level |
| Platelet count | ≥50×10^3^/µL  <75×10^3^/µL | Dose not decreased |
|  | <5.0×10^3^/µL | Dose decreased by one level |
| Serum creatinine | >1.2 mg/dL | Dose decreased by one level |
| Infection | Fever (≥38.0°C) with suspected infection | Dose not decreased |
| Gastrointestinal symptoms (diarrhea, stomatitis, nausea, vomiting, anorexia) | Grade 2 | Dose not decreased |
|  | ≥Grade 3 | Dose decreased by one level |
| Other nonhematologic symptoms | ≥Grade 3 | Dose decreased by one level |

Table 7.6.2. Reduced dose levels of TS-1

| Initial dose | Reduced dose levels | |
| --- | --- | --- |
|  | －1 level | －2 levels |
| 60 mg/day^※^  80 mg/day  100 mg/day  120 mg/day | 50 mg/day  60 mg/day^※^  80 mg/day  100 mg/day | －  50 mg/day  60 mg/day^※^  80 mg/day |

※: Morning, 40 mg; evening, 20 mg

## 7.7. Modification of treatment schedule for TS-1

### 7.7.1. Criteria for withdrawal of TS-1 and resumption of next course

1) If treatment with TS-1 is withdrawn for 7 consecutive days or longer because of factors such as adverse events, the course is discontinued, and treatment is resumed as the next course.

Example: If treatment with TS-1 is withdrawn on day 22 and resumed on day 29, that day is designated as day 1 of the next course of treatment.

2) If the withdrawal period of TS-1 is within 6 days, treatment is resumed as the same course. However, treatment with TS-1 is not given beyond the previously designated treatment schedule.

Example: If TS-1 is scheduled to be given for 4 weeks followed by a 2-week rest, TS-1 is not given after day 29.

3) The next course of treatment with TS-1 is resumed after confirming that the patient meets the conditions of “Table 7.5.1. Criteria for starting a course of treatment with TS-1 (course 2 onwards).”

Table 7.7.1. Criteria for resuming treatment with TS-1

| TS-1 rest period | Handling of the time of resuming treatment | Criteria for resuming treatment |
| --- | --- | --- |
| Within 6 days | Treatment resumed as same course  (Treatment within the initial treatment schedule) | “Table 7.4.1.2. Criteria for resuming treatment with TS-1 within a course” |
| 7 days or longer | Treatment resumed as next course | "Table 7.5.1. Criteria for starting a course of treatment with TS-1 (course 2 onwards)" |

Criteria for resuming course confirmed

Conflict with criteria for withholding course of TS-1

Withdrawal of treatment for ≥7 days

TS-1

TS-1

Day1

Day1

Course n+1

Course n

Figure 7.7.1. Conceptual diagram of resuming the next course when treatment has been discontinued

**7.7.2. Switching to treatment with TS-1 for 2 weeks followed by a 7-day rest**

1) If the study director or attending physician judges that treatment with TS-1 for 2 weeks followed by a 7-day rest is difficult to continue because of adverse events or other factors that develop after day 15 of the previous course from course 2 onwards, the treatment regimen of TS-1 can be switched to 2 weeks of treatment followed by a 7-day rest.

2) In accordance with "Table 7.2.1. Daily dose of TS-1," treatment with TS-1 is started after breakfast on day 1. TS-1 is given orally twice daily (after breakfast and dinner) until after dinner on day 14, followed by a 7-day rest.

3) Even if treatment is withheld during a course of treatment or a patient forgets to take their medication, treatment is not be performed after day 15. However, if treatment cannot be given after breakfast on day 1, treatment is started from after dinner and is continued until after breakfast on day 15.

4) A treatment period of 3 weeks is regarded as 1 course.

## 7.8. Modification of the dose and treatment schedule for trastuzumab

### 7.8.1. Permissible modification of the day of treatment with trastuzumab

If treatment with trastuzumab cannot be given on the scheduled day because of factors other than adverse events, such as the patient’s ability to come to the hospital or holidays, treatment will be given as described below.

1) The acceptable margin of treatment with trastuzumab is shown in "Table 7.8.1. Acceptable range of the day of treatment with trastuzumab."

Table 7.8.1. Acceptable range of the day of treatment with trastuzumab

| Scheduled day of treatment with trastuzumab | Treatment margin | Acceptable range of the day of treatment with trastuzumab |
| --- | --- | --- |
| Day 1 | + 2 days | Day 1 to day 3 |
| Day 22 | ± 2 days | Day 20 to day 24 |

2) Irrespective of the presence or absence of treatment with trastuzumab, the next dose is given at a 3-week interval in accordance with the initial treatment schedule (on day 1 and day 22 of each course).

Example: If trastuzumab cannot be given as scheduled on day 1, and trastuzumab is given on day 3, the scheduled day of the next dose is day 22, which is 3 weeks after day 1, the originally scheduled day for treatment.

3) If trastuzumab cannot be given within 28 days after the previous dose of trastuzumab and is given on day 29 or subsequently, trastuzumab is given in an initial dose of 8 mg/kg.

Example: If trastuzumab is given on day 1, and the next dose is given on day 30 or subsequently, a dose of 8 mg/kg is administered.

### 7.8.2. Treatment schedule for trastuzumab when treatment with TS-1 is withheld or discontinued

If treatment with TS-1 is withheld or discontinued for some reason, trastuzumab is administered as described below.

1) Even if treatment with TS-1 is withheld or discontinued, trastuzumab is given at a 3-week interval.

Example: If trastuzumab is given on day 22, and the criteria for starting the next course of treatment with TS-1 are not met on day 43 (the day scheduled for initiation of the next course of treatment), trastuzumab alone is given on day 43.

2) When treatment with TS-1 is resumed, trastuzumab is given on day 1 of the resumed course of treatment and then given at 3-week intervals, starting from day 1.

# 8. Criteria for discontinuing the protocol treatment

If a patient meets any of the following criteria, the protocol treatment is discontinued.

1) The protocol treatment could not be started for more 14 days after enrollment.

2) Progressive disease (PD) appeared after the start of treatment.

3) Even after the dose of TS-1 was decreased to the lowest level, adverse events requiring a further reduction in dosage developed.

4) The patient did not meet "Table 7.5.1. Criteria for starting a course of treatment with TS-1 (course 2 onwards)" because of adverse event that developed during the previous course of treatment, even after more than 28 days had elapsed since the final day of treatment with TS-1.

5) The study director or attending physician judges that treatment is difficult to continue because of the development or exacerbation of complications.

6) The study director or attending physician judges that treatment is difficult to continue because of adverse events.

7) The patient requests to the protocol treatment to be discontinued.

8) The patient is transferred to another hospital.

9) The patient dies (In the case of death, the date of death is considered the date of discontinuing treatment.)

10) The patient retracts informed consent.

11) The patient is found to be ineligible after enrollment.

12) The study director or attending physician considers treatment continuation to be inappropriate.

If the study treatment is discontinued, the day of discontinuation is defined as the day on which treatment was terminated by the attending physician, not the day of the development of adverse events that led to the termination of treatment.
